# Validation of multiple sclerosis diagnoses in the Swedish National Patient Register

**DOI:** 10.1007/s10654-019-00558-7

**Published:** 2019-09-06

**Authors:** Chantelle Murley, Emilie Friberg, Jan Hillert, Kristina Alexanderson, Fei Yang

**Affiliations:** 1 Division of Insurance Medicine, Department of Clinical Neuroscience, Karolinska Institutet, 171 77 Stockholm, Sweden; 2 Division of Neurology, Department of Clinical Neuroscience, Karolinska Institutet, 171 77 Stockholm, Sweden

**Keywords:** Validation studies, Electronic health records, Multiple sclerosis, Sick leave, Administrative data, Swedish National Patient Register

## Abstract

Population-based registers are widely used in epidemiological studies. We aimed to estimate the validity of multiple sclerosis (MS) diagnoses registered in the Swedish National Patient Register (NPR) by two sequential register-based case-definition algorithms. Prevalent MS patients aged 16–64 years were identified from the in- and specialised out-patient NPR in 2001–2013, using International Classification of Diseases code G35. These identified MS diagnoses were validated through two sequential register-based case-definition algorithms, as the ‘gold-standard’ reference, by linking individual-level data longitudinally to other nationwide registers. The primary algorithm first sought to corroborate the MS diagnoses with MS-specific information in other nationwide registers. The exploratory secondary algorithm identified individuals with MS-related information in other registers and those who were unable to be followed sufficiently. Through multi-register linkage, we estimated the number of confirmed and uncertain individuals with an MS diagnosis recorded in the NPR. A total of 19,781 individuals (mean age at first visit 45.2 years; 69.5% women) had at least one MS diagnosis recorded in the NPR during 2001–2013. Using the two case-definition algorithms, 92.5% (n = 18,291) of the MS diagnoses recorded in the NPR were confirmed, while 7.5% (n = 1490) remained uncertain. Our findings indicate that a very high percentage of patients coded with an MS diagnosis in the Swedish NPR actually have MS, and supports the use of the NPR as a viable source to identify individuals with an MS diagnosis for population-based research. This exploratory methods paper suggests an alternative novel method to verify individuals’ diagnoses in register-based settings.

**Electronic supplementary material:**

The online version of this article (10.1007/s10654-019-00558-7) contains supplementary material, which is available to authorized users.

## Background

The Swedish nationwide population-based registers are widely used in epidemiological studies, providing a less resource demanding data source than patient records to establish representative scientific results [1–4]. Nonetheless, administrative registers may be subject to diagnostic and coding errors [5]. Individual-level linkage across the different registers can be used to corroborate the information contained within a single register. The National Patient Register (NPR), a principal data source for research [4, 5], contains records of healthcare visits in Sweden with diagnoses registered by the International Classification of Diseases (ICD) codes. The validity of the complete NPR is high [5], but knowledge of data quality is lacking for some specific diseases, such as multiple sclerosis (MS).

MS is a neurodegenerative disease that typically affects younger adults, causing increasing levels of cognitive and physical impairment as the disease progresses [6–8]. Sweden has an especially high prevalence [7]; with an estimated all-age nationwide MS prevalence of 189 per 100,000 [9]. Despite that MS diagnoses are now being set earlier and more accurately, no single clinical feature or diagnostic test can positively diagnose MS [10–12]. This clinical context may reduce our certainty of the recorded MS diagnoses due to the risk of misdiagnosis between MS and other degenerative neurological diseases, a risk which has been suggested to still exist with the 2017 update of the McDonald criteria [10, 13]. Therefore, it has been questioned to what extent an MS diagnosis is incorrectly assigned and recorded in administrative records during the period investigating whether a patient has MS [10, 13–18].

Besides the NPR, several other Swedish nationwide registers contain MS-specific information (i.e., MS ICD codes or medications), which could be used to identify MS cases. Accordingly, we aimed to assess the validity of MS diagnoses recorded in the NPR with a novel method using information linked from other nationwide registers in two sequential register-based case-definition algorithms.

## Methods

This validation study evaluates the NPR as a relevant source for MS studies by comparing the records of all individuals aged 16–64 with an MS code during 1 January 2001–31 December 2013 in the NPR against other register-based MS records, through two sequential register-based case-definition algorithms as the ‘gold-standard’ reference.

### Data sources

The unique personal identity number assigned to all residents in Sweden enabled individual-level linkages across six nationwide registers [19], administered by the following four authorities:

- **National Board of Health and Welfare**: *The National Patient Register* (NPR) includes information about healthcare visits, including main and all side diagnoses coded according to the Swedish version of the ICD at time of visit [20]. The NPR has had nationwide coverage since 1987 of in-patient admissions and of out-patient specialist visits since 2001 [5]. *The Swedish Prescribed Drug Register* (SPDR) contains nationwide records of dispensed medication prescriptions with Anatomical Therapeutic Classification (ATC) codes since 1 July 2005 [21]. *The Cause of Death Register* (CDR) contains nationwide information on the date, underlying and contributory causes of death recorded by ICD codes since 1961 [22].
- **Karolinska University Hospital**: *The Swedish MS Register* (SMSReg) is a nationwide but voluntary MS-specific clinical register that is used for nationwide pharmacological surveillance and for enhancing quality of care [23, 24]. SMSReg contains comprehensive clinical information [25] for included patients diagnosed with MS to support clinical decision-making, including retrospective information predating the SMSReg’s establishment in 2001 [26]. The accuracy and completeness of the clinical data has been recently estimated to be of value for future studies [25].
- **Social Insurance Agency**: *The Micro*-*Data for Analysis of the Social Insurance System* (MiDAS) contains information on sickness absence (since 2005) and disability pension benefits (since 1994) (dates and diagnoses (by ICD codes)) [27].
- **Statistics Sweden**: *The Longitudinal Integration Database for Health Insurance and Labour Market Studies* (LISA), comprises annual socio-demographic information on all people registered as living in Sweden [28, 29].

### Study population

**Fig. 1:**
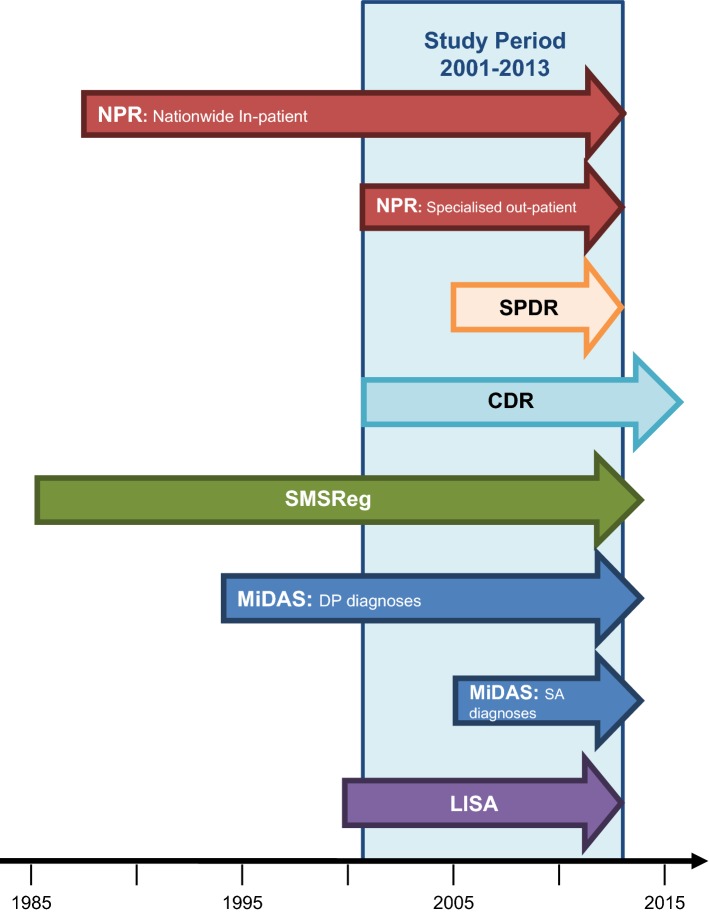
Data coverage and availability from the nationwide register sources in relation to the study period. *Notes* The study period refers to dates to identify MS cases from the Swedish National Patient Register (NPR) among people aged 16–64 at the time of the visit. Six nationwide registers containing individual-level data were used. The NPR (red arrows) contains information healthcare visits according to ICD codes, but with different coverage dates with regards to the healthcare setting. MS healthcare visits in both the in-patient (nationwide since 1987) and specialised out-patient (included 2001) healthcare settings until 31 December 2013 were included. The Swedish Prescribed Drug Register (SPDR) (orange arrow) contains information of the nationwide records of dispensed medication prescriptions with Anatomical Therapeutic Classification (ATC) codes since 1 July 2005 and were available until 31 December 2013. The Cause of Death Register (CDR) (aqua arrow) contains nationwide information since 1961 on the date and underlying cause of death, including contributory causes, according to ICD codes. ICD codes were not available after 2016. The nationwide voluntary clinical quality register, Swedish MS Register (SMSReg) (green arrow), was established in 2001 and contains comprehensive clinical information of MS-related care for the included patients, including retrospective information predating the SMSReg’s creation from selected neurology clinics and was available until September 2014. Micro-Data for Analysis of the Social Insurance System (MiDAS) (blue arrows) has coverage of diagnoses for disability pension (DP) benefits from 1994 and diagnoses for sickness absence (SA) benefits from 2005, and was available until 2014. Longitudinal Integration Database for Health Insurance and Labour Market Studies (LISA) (purple arrow) contains annual individual-level information about the socio-demographics of the total population registered as resident in Sweden, as of 31 December, and was used for the years 2000–2013. *ATC* Anatomical Therapeutic Classification, *CDR* Cause of Death Register, *DP* disability pension, *ICD* International Classification of Diseases, *LISA* Longitudinal Integration Database for Health Insurance and Labour Market Studies, *MiDAS* Micro-Data for Analysis of the Social Insurance System, *MS* multiple sclerosis, *NPR* National Patient Register, *SA* sickness absence, *SPDR* Swedish Prescribed Drug Register, *SMSReg* Swedish MS Register

All individuals with a main or side diagnosis of MS (ICD-10: G35) recorded when having a healthcare visit in 2001–2013, aged 16–64 years, were identified in the NPR (n = 19,781). From the date of first MS diagnosis in 2001–2013 in the NPR, both retrospective and prospective information was obtained from the above mentioned six registers, in order to confirm the MS diagnosis. If it was not possible to confirm the MS diagnosis, the individual was followed until emigration, death, or end of data extraction of each register (varies between 2013 and 2016), whichever came first (Fig. 1).

### Statistical analyses

We first described the MS patients in terms of frequencies and percentages by: sex (women/men); healthcare setting (in-/out-patient); whether MS was the main diagnosis for the first record with an MS diagnosis during 2001–2013 (yes/no); number of visits with an MS diagnosis recorded in the NPR ever (< 3/≥ 3); whether in the NPR prior to 2001 with an MS diagnosis (yes/no); whether died before 31 December 2013 (yes/no); and age at first record with MS in the NPR during 2001–2013.

The annual MS prevalence (total and sex-specific) were estimated from 2001 to 2013 of all individuals aged 16–64 at the time of MS diagnosis according to the NPR. The prevalence estimates were expressed per 100,000 of the total population in Sweden aged 16–64, identified in LISA for the respective year.

#### Validation

Two sets (primary and secondary) of register-based case-definition algorithms were constructed by the multidisciplinary research group for this study to validate MS diagnoses recorded in the NPR with the extensive register data available. The algorithms cross-checked the MS diagnoses at an individual-level with specific information on MS from all available nationwide register sources in Sweden, including prior to and after 2001–2013 (Fig. 1). These algorithms, applied in a sequential manner, were thought to be equivalent to a diagnostic test to confirm the registered diagnoses [30]. Accordingly, the MS diagnoses recorded in the NPR were treated as ‘provisional’ MS until validated as ‘confirmed’ MS as per the algorithms.

**Fig. 2:**
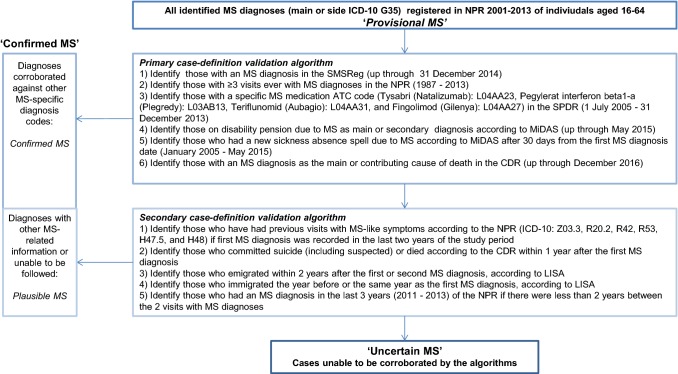
Primary and secondary case-definition algorithms which together form the ‘gold standard’ to identify the confirmed MS diagnoses registered in the Swedish National Patient Register (NPR) by validating against individual-level information from other register data sources. *Notes* Given that patients with clinically isolated syndrome (CIS) should be kept as uncertain MS, and may be treated with a subset of DMTs, the following DMTs and ATC codes were not included in the algorithm: Interferon beta 1-a: L03AB07, Interferon beta 1-b: L03AB08, and Glatiramercetat: L03AX13. *ATC* Anatomical Therapeutic Classification, *CDR* Cause of Death Register, *CIS* clinically isolated syndrome, *DMT* disease modifying therapies, *ICD* International Classification of Disease, *LISA* Longitudinal Integration Database for Health Insurance and Labour Market Studies, *MiDAS* Micro-Data for Analysis of the Social Insurance System, *MS* multiple sclerosis, *NPR* National Patient Register, *SPDR* Swedish Prescribed Drug Register, *SMSReg* Swedish MS Register

Figure 2 displays the primary and secondary sets of case-definition algorithms, including the order of the steps identifying *confirmed* MS and the ‘gold-standard’ data source used [1]. The primary algorithm identified MS-specific codes (ICD-10 G35, ICD-9 340, or specific ATC codes for MS medications) in other registers in six steps. In the first step of this algorithm, individuals with clinical MS information, entered by their treating neurologist, in the SMSReg were identified. The secondary case-definition was then applied to the remaining *provisional* MS diagnoses after completing the primary algorithm. This exploratory algorithm matched to other MS-related information (MS-like symptom ICD codes), and sought to identify individuals who were unable to be sufficiently followed in the data to confirm the diagnosis as ‘plausible’ MS. The individuals who remained at the end of the last step in each set of case-definition algorithms were classified as ‘uncertain’ MS (i.e., patients who received at least one MS diagnosis recorded in the NPR, but without other register-based information corroborating the diagnosis). The *confirmed* MS are those identified according to both case-definition algorithms, i.e., had other register-based MS information supporting their MS diagnosis in the NPR. We then plotted the proportions of *confirmed*, *plausible*, and *uncertain* MS by the year of first MS record in the NPR.

Finally, in order to estimate the influence of the *uncertain* MS cases in the NPR if included in study populations, we profiled the descriptive characteristics of those remaining as *uncertain* MS after each set of algorithms and those *confirmed* MS (including *plausible* MS diagnoses). Similarity of the characteristics of *uncertain* MS after the secondary algorithm and those of *confirmed* MS were tested using Chi square tests. The mean number of years between the first and second MS record in the NPR with 95% confidence intervals (CI) were calculated. All analyses were conducted in SAS v.9.4.

### Compliance with ethical standards

This project was approved by the Regional Ethical Review Board of Stockholm in accordance with the Declaration of Helsinki.

## Results

Overall, 19,781 individuals (69.5% women) with MS diagnoses recorded in the NPR when aged 16–64 were identified in 2001–2013. In all, 81.7% (n = 16,158) were first identified from specialised out-patient visits and 89.7% (n = 17,738) had MS as the main diagnosis. They together had 308,761 visits with MS as a listed diagnosis during 2001–2013.

**Fig. 3:**
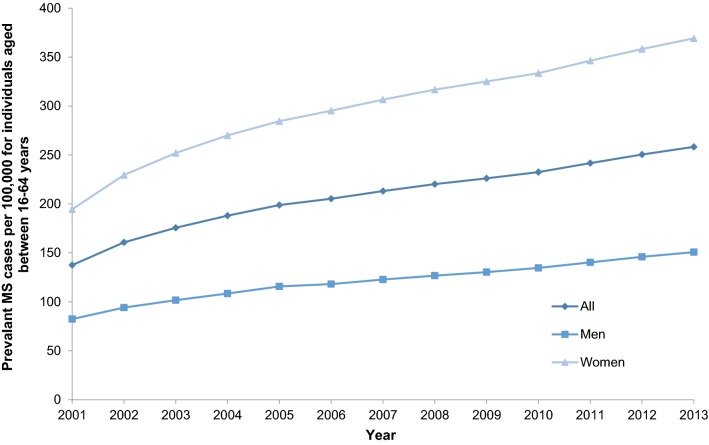
Prevalence of MS per 100,000 individuals aged 16–64 in 2001–2013, identified in the Swedish National Patient Register. *MS* multiple sclerosis

The nationwide prevalence of MS according to the NPR in the population aged 16–64 years steadily increased over the study period (Fig. 3). In 2013, the estimated prevalence was 258.3 (women 369.1; men 150.8) per 100,000. Women had a higher prevalence than men throughout the study.

Of the identified individuals, 92.5% (n = 18,291) had their diagnosis confirmed by the validation algorithms (Table 1). The primary algorithm confirmed the majority of diagnoses (17,922; 90.6%), with fewer corroborated by the secondary algorithm (369; 1.9%). We classified 1490 (7.5%) individuals as *uncertain* MS after cross-checking both case-definition algorithms. After the first two steps of the primary algorithm, MS diagnosis in the SMSReg and ≥ 3 visits due to MS recorded in the NPR, 89.4% (n = 17,692) of the *provisional* MS diagnoses were confirmed.

**Table 1:** Steps in the process to validate the MS diagnoses recorded in the Swedish National Patient Register (NPR) (2001–2013) (n = 19,781)^a^ by two sequential register-based case-definition algorithms

	N identified (i.e., *confirmed *MS)	N remaining (i.e., *uncertain* MS)
Primary case-definition algorithm		
(1) Identify those with an MS diagnosis in the SMSReg (up through 31 December 2014)	14,033	5748
(2) Identify those with ≥ 3 visits ever with MS diagnoses in the NPR (1987–2013)	3659	2089
(3) Identify those with a specific MS medication ATC code (Tysabri (Natalizumab): L04AA23, Pegylerat interferon beta1-a (Plegredy): L03AB13, Teriflunomid (Aubagio): L04AA31, and Fingolimod (Gilenya): L04AA27) in the SPDR (1 July 2005–31 December 2013)	6	2083
(4) Identify those on disability pension due to MS as main or secondary diagnosis according to MiDAS (up through May 2015)	158	1925
(5) Identify those who had a new sickness absence spell due to MS according to MiDAS after 30 days from the first MS diagnosis date (January 2005–May 2015)	51	1874
(6) Identify those with an MS diagnosis as the main or contributing cause of death in the CDR (up through December 2016)	15	1859
**Subtotals from the primary case-definition algorithm**	**17,922**	**1859**
Secondary case-definition algorithm		
(1) Identify those who have had previous visits with MS-like symptoms according to the NPR (ICD-10: Z03.3, R20.2, R42, R53, H47.5, and H48) if the first MS diagnosis was recorded in the last 2 years of the study period^b^	177	1682
(2) Identify those who committed suicide (including suspected) or died according to the CDR within 1 year after the first MS diagnosis	33	1649
(3) Identify those who emigrated within 2 years after the first or second MS diagnosis, according to LISA	28	1621
(4) Identify those who immigrated the year before or the same year as the first MS diagnosis, according to LISA	15	1606
(5) Identify those who had an MS diagnosis in the last 3 years (2011–2013) of the NPR if there were less than 2 years between the 2 visits with MS diagnoses	116	1490
**Subtotals from the secondary case-definition algorithm**	**369**	**1490**
**Total**	**18,291**	**1490**

*ATC* Anatomical Therapeutic Classification, *CDR* Cause of Death Register, *ICD* International Classification of Diseases, *LISA* Longitudinal Integration Database for Health Insurance and Labour Market Studies, *MiDAS* Micro-Data for Analysis of the Social Insurance System Register, *MS* multiple sclerosis, *NPR* National Patient Register, *SPDR* Swedish Prescribed Drug Register, *SMSReg* Swedish MS Register

^a^All individuals with a record of an MS diagnosis registered during 1 January 2001 and 31 December 2013 as per ICD-10 (G35) in the Swedish National Patient Register and aged 16–64 at first diagnosis in the study period

^b^Z03.3: Observation for suspected nervous system disorder, R20.2: Paraesthesia of skin, R42: Dizziness and giddiness, R53: Malaise and fatigue, H47.5: Disorders of other visual pathways, and H48: Disorders of optic nerve and visual pathways in diseases classified elsewhere

The profiles of the *confirmed* and *uncertain* MS are presented in Table 2. The mean number of years between the first and second visit (2001–2013) in the NPR for the *uncertain* MS were 0.85 (95% CI 0.70–1.00) after the primary case-definition algorithm and 1.01 (95% CI 0.78–1.24) after the secondary case-definition algorithm. Of the individuals with *confirmed* MS (n = 18,291), 16,527 (90.4%) had MS as the main diagnosis and 14,921 (81.6%) first presented to out-patient healthcare. Of the *uncertain* MS, most of them had only one visit due to an MS diagnosis recorded in the NPR (78.5%, n = 1169) (not presented in the table).

**Table 2:** Characteristics of individuals identified during 1 January 2001–31 December 2013 with an MS diagnosis registered in the Swedish National Patient Register (NPR)^a^ when aged 16–64, according to the register-based case-definition algorithms

	*Provisional* MS diagnoses in the NPR		*Uncertain* MS as per Primary case-definition algorithm		*Uncertain* MS as per Secondary case-definition algorithm		*Confirmed* MS as per both case-definition algorithms		χ^2^ test
	n = 19,781		n = 1859^b^		n = 1490^b^		n = 18,291		*p* value^c^
	n	%	n	%	n	%	n	%	
Sex									< 0.001
Women	13,743	69.5	1224	65.8	970	65.1	12,773	69.8	
Men	6038	30.5	635	34.2	520	34.9	5518	30.2	
Healthcare setting of first recorded visit in the NPR with MS									0.166
In-patient Register	3623	18.3	322	17.3	253	17.0	3370	18.4	
Out-patient Register	16,158	81.7	1537	82.7	1237	83.0	14,921	81.6	
Main diagnosis as MS for the first recorded visit	17,738	89.7	1520	81.8	1211	81.3	16,527	90.4	< 0.001
Number of visits in the NPR with MS ever^d^									< 0.001
< 3	2834	14.3	1859	100.0	1490	100.0	1344	7.3	
≥ 3	16,947	85.7	^c^	^c^	^c^	^c^	16,947	92.7	
In the NPR prior to 1 January 2001 with MS	4399	22.2	29	1.6	28	1.9	4371	23.9	< 0.001
Died (all-cause) during 2001–2013	1806	9.1	96	5.2	65	4.4	1741	9.5	< 0.001
Age at first record with MS in the NPR during 2001–2013									
16–20	508	2.6	42	2.26	31	2.08	477	2.61	
21–25	1072	5.4	91	4.90	73	4.90	999	5.46	
26–30	1813	9.2	168	9.04	127	8.52	1686	9.22	
31–35	2138	10.8	202	10.87	155	10.40	1983	10.84	
36–40	2397	12.1	201	10.81	174	11.68	2223	12.15	
41–45	2686	13.6	219	11.78	173	11.61	2513	13.74	
46–50	2621	13.3	234	12.59	181	12.15	2440	13.34	
51–55	2731	13.8	243	13.07	201	13.49	2530	13.83	
56–60	2451	12.4	273	14.69	224	15.03	2227	12.18	
61–64	1364	6.9	186	10.01	151	10.13	1213	6.63	

*ICD* International Classification of Diseases, *MS* multiple sclerosis, *NPR* National Patient Register

^a^Identified by ICD-10 code G35 registered as the main or side diagnosis for in-patient and specialised out-patient care in the NPR. Inclusion criteria: All individuals with a record of a prevalent MS diagnosis registered in the Swedish NPR and aged 16–64 years at the time of the first code appearing during the study period 1 January 2001 and 31 December 2013, n = 19,781

^b^n = 369 individuals were *plausible* MS as per the second case-definition algorithm and thus many individuals in the primary and secondary algorithm groups overlap

^c^Significantly different proportions between the *confirmed* MS (both algorithms) and *uncertain* MS as per secondary algorithm, as tested by Chi square tests with alpha set at 0.05

^d^Step 3 of the primary-case definition algorithm was to identify individuals with > 3 visits ever recorded in the NPR with MS as *confirmed* MS

A peak in first time NPR-registered MS diagnoses was observed when out-patient healthcare was included in 2001 (Online Resource 1).

## Discussion

In this study we identified all individuals aged 16–64 with MS as a main or side diagnosis in the Swedish NPR during 2001–2013. We compared these provisional MS diagnoses against individual-level MS-specific information in other nationwide registers. With this novel method, we confirmed that 92.5% of the NPR-registered MS diagnoses could be validated after the two sequential register-based case-definition algorithms. Our findings support the NPR as a valuable source to identify MS cases in epidemiological studies.

The majority of provisional and confirmed MS diagnoses first presented in out-patient settings, with MS as the main diagnosis. A slightly higher percentage of the *uncertain* MS diagnoses were a side diagnosis. The spike in the number of registered MS diagnoses in 2001 corresponds with the inclusion of the specialised out-patient visits, and tapers off by 2004 to reasonably consistent annual numbers of first time identified diagnoses for the remaining study years. This trend suggests caution is required when identifying newly diagnosed MS cases from the NPR prior to 2004, i.e., the first years of including out-patient visits in the NPR.

The first two steps of the primary case-definition algorithm (MS diagnosis in the SMSReg and ≥ 3 visits with MS ever in the NPR) confirmed the majority of provisional MS diagnoses. The exploratory secondary case-definition algorithm identified a small number of plausible MS diagnoses, predominantly in the later years, with insufficient follow-up time to reasonably identify MS information in the registers. The individuals with *uncertain* MS after both case-definition algorithms could plausibly represent patients who were initially suspected of having MS, but the diagnosis was never confirmed, or simply due to administrative error. In total, 78.5% of the *uncertain* MS only had one visit ever due to MS.

A previous cross-referencing of the SMSReg to NPR by the National Board of Health and Welfare found that only 4% of MS entries in the NPR in 2008 were coded as ‘possible MS’ in the SMSReg [26], suggesting high validity of MS diagnoses recorded in the NPR [26]. Accordingly, when selecting a study population, the voluntary SMSReg may underestimate the MS population, especially for particular geographical regions [26], and we, based on this study, add that the mandated NPR may slightly overestimate the MS population (< 10%). Further studies are needed to elucidate occurrence of this.

Our prevalence estimates of MS for 16–64 year olds were higher than an all-age estimate of 189 per 100,000 at 31 December 2008 with MS identified in either the SMSReg or NPR [9]. The improved diagnostic criteria, better awareness, more efficacious disease modifying therapies (DMTs), and ultimately extended survival could be a potential explanation [6, 7]. The high prevalence of MS among working-aged individuals and increasing treatment options necessitates representative real-world results [6], which the NPR enables [1, 2].

Strengths of this study include the numerous nationwide registers with MS-specific information available to corroborate the MS diagnoses in the NPR. Usually, a ‘gold-standard’ of disease is derived from clinical data, albeit for a sample of the total population. Instead, we used a novel method of register-based algorithms linking individual-level register data, including MS-specific clinical information from the SMSReg [25], to determine the validity of all identified MS cases in Sweden in 2001–2013. This study included years both before and after identification in the NPR, a design consistent with the chronic and progressive nature of MS [31] and treatment guidelines for annual healthcare visits for individuals with MS or clinically isolated syndrome (CIS) [32].

One limitation was that the case-definition algorithms were limited to the different coverage dates of the contributing registers. For example, the SPDR, which was available for July 2005–December 2013 and only covered dispensed prescribed drugs, therefore, excluding both indented drugs administrated at clinics and DMTs that became available after 2013. Further, both MS and CIS patients may be prescribed interferon beta therapies [32], therefore, these ATC codes could not be used to confirm MS diagnoses.

In conclusion, the certainty of MS diagnoses for patients aged 16-64 in the NPR is very high according to this validation using register-based algorithms. These findings strengthen the notion that the Swedish NPR is a valuable source for MS populations in nationwide epidemiological studies.

## Electronic supplementary material

Below is the link to the electronic supplementary material.

Supplementary material 1 (PDF 13 kb)

## Data Availability

Not applicable. The data cannot be made publically available due to privacy regulations. The data used in this study is administered by the Division of Insurance Medicine, Karolinska Institutet. According to the General Data Protection Regulation, the Swedish law SFS 2018:218, the Swedish Data Protection Act, the Swedish Ethical Review Act, and the Public Access to Information and Secrecy Act, these types of sensitive data can only be made available, after legal review, for researchers who meet the criteria for access to this type of sensitive and confidential data. Readers may contact Professor Kristina Alexanderson (kristina.alexanderson@ki.se) regarding the data.
