# Supplementary material for: Validation of multiple sclerosis diagnoses in the Swedish National Patient Register
Source: Eur J Epidemiol. 2019 Sep 6;34(12):1161–9. doi: 10.1007/s10654-019-00558-7 (PMC7010617; doi:10.1007/s10654-019-00558-7)
Supplement: Supplementary file 1 — Supplementary material 1 (PDF 13 kb) [file 10654_2019_558_MOESM1_ESM.pdf]

Validation of multiple sclerosis diagnoses in the Swedish National Patient Register

European Journal of Epidemiology

Chantelle Murley[1], Emilie Friberg[1], Jan Hillert[2], Kristina Alexanderson[1], Fei Yang[1]

[1] Division of Insurance Medicine, Department of Clinical Neuroscience, Karolinska Institutet, SE-171 77 Stockholm, Sweden.

[2] Division of Neurology, Department of Clinical Neuroscience, Karolinska Institutet, SE-171 77 Stockholm, Sweden.

Corresponding author: Chantelle Murley: chantelle.murley@ki.se

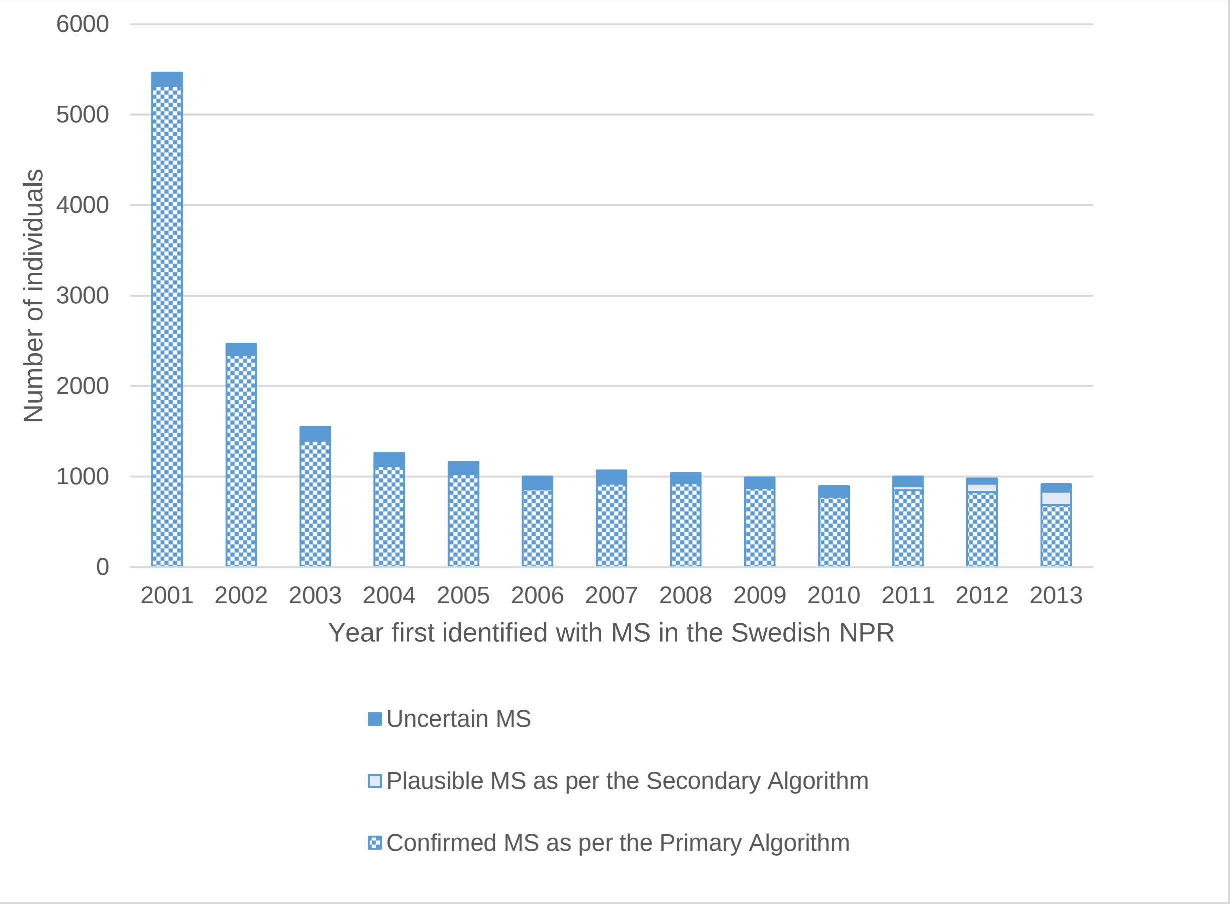

Online Resource 1 Proportions of the registered MS cases per year of first appearance in the Swedish National Patient Register (NPR) during 2001-2013 which are confirmed or remained uncertain after both of the two case-definition algorithms

Notes: Year the individual first appears in the NPR within the study period of 2001-2013.
Abbreviations: MS: multiple sclerosis, NPR: National Patient Register.
